# Metamorphic turnover at 2 Ga related to two-stage assembly of Columbia

**DOI:** 10.1038/s41598-024-56691-1

**Published:** 2024-03-18

**Authors:** Silvia Volante, Uwe Kirscher

**Affiliations:** 1 Structural Geology and Tectonics Group, Geological Institute, Department of Earth Sciences, ETH Zürich, Zürich, Switzerland; 2 Earth Dynamics Research Group, The Institute for Geoscience Research (TIGeR), School of Earth and Planetary Sciences, Curtin University, Bentley, WA Australia

**Keywords:** Solid Earth sciences, Geodynamics, Geology, Palaeomagnetism, Petrology, Tectonics

## Abstract

Understanding the stabilization of cratons and how this is related to the onset of plate-tectonics is among the most important questions in geoscience. The assembly of Earth’s first supercontinent Columbia represents the first lines of evidence for a global subduction network, when the oldest, deep subduction-related rocks have been reported. We combine the low-, intermediate- and high-T/P global metamorphic record with the two-stage assembly of the Nuna-Columbia supercontinent to address the significance of the oldest “cold” rocks (low-T/P) and the related emergence of bimodal metamorphic belts. For this purpose, we analyse two examples from Laurentia (including Greenland) and Australia between 2.0–1.8 Ga and 1.8–1.6 Ga. Two main observations are: (i) a first-stage (2.0–1.8 Ga) amalgamation of the megacontinent Nuna (precursor to Columbia) is characterized by bimodal metamorphism along major mobile belts suturing the megacontinent’s center. In contrast, a second-stage (1.8–1.6 Ga) is dominated by the formation of soft collisional orogens during the final Columbia supercontinent assembly, recording intermediate- to high-T/P metamorphism; (ii) the metamorphic signature of the two assembly stages, featuring low- and intermediate-T/P rocks during Nuna assembly followed by their near absence during Columbia amalgamation, contrasts with the thermobaric ratios recorded by the Phanerozoic Gondwana-Pangea assembly, where intermediate and low-T/P rocks dominated the final stage of Pangea amalgamation. This discrepancy may signify substantial changes in intraplate metamorphism and minor rearrangements during Columbia assembly compared to major continent–continent collisions, such as the Appalachian-Variscan Orogen as well as production and fast exhumation of high- to ultra-high-pressure rocks during the assembly of the supercontinent Pangea. Furthermore, the variation of thermobaric ratios aligns with the concept of a two-stage mega-supercontinent formation, emphasizing differences between the potentially oldest and youngest supercontinent cycles.

## Introduction

**Figure 1:**
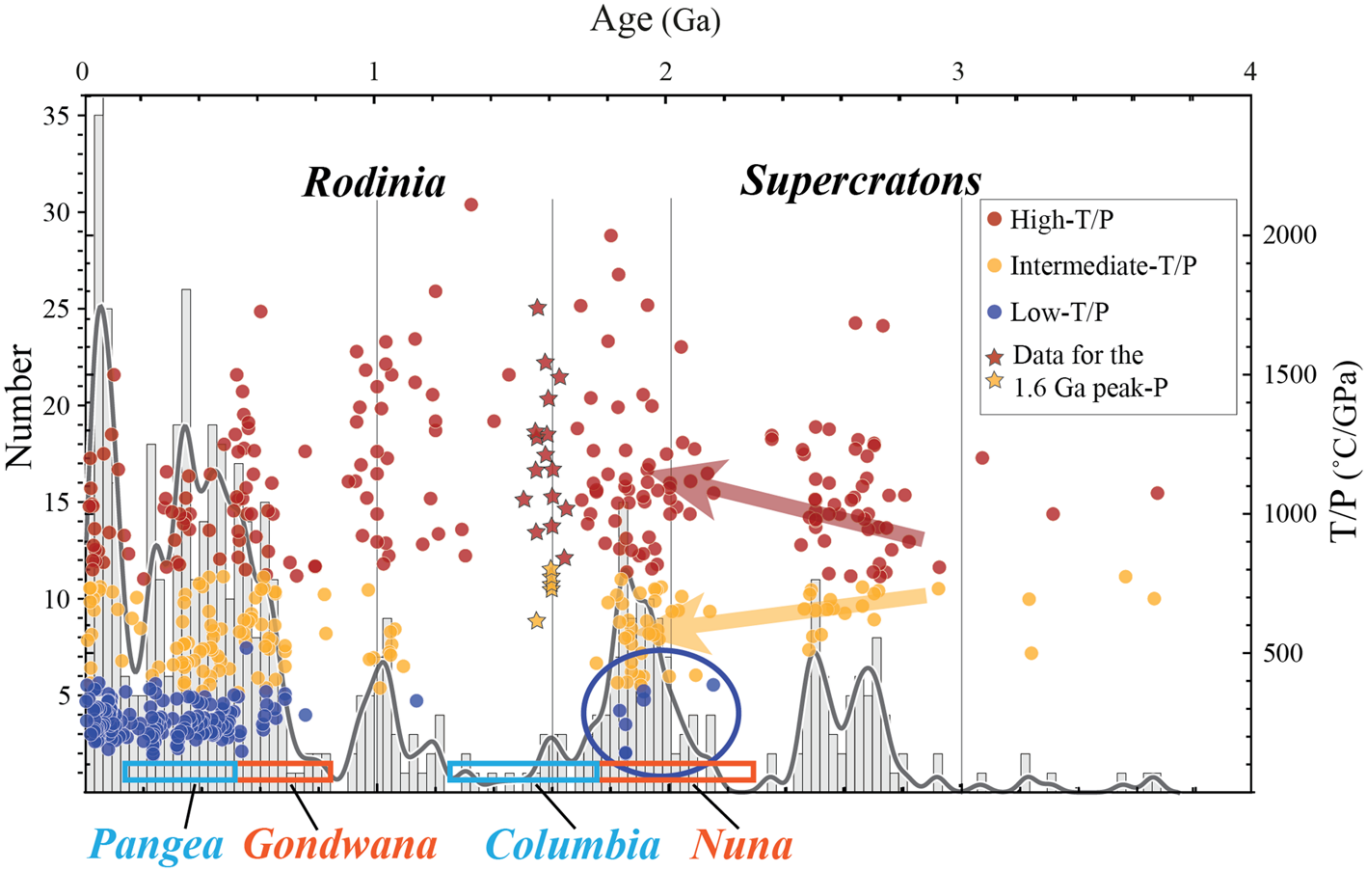
Metamorphic *T/P* (C°/GPa) records versus ages (Ga) modified after Brown and Johnson^71^, where three types of metamorphism are identified: high-*T/P* (red), intermediate-*T/P* (orange) and low-*T/P* (blue). Bimodal metamorphism (marked by the red and orange arrows) and associated appearance of the first low-*T/P* metamorphic rocks (blue circles) can be related to the first supercontinent cycle with the assembly of the megacontinent Nuna, followed by the supercontinent Columbia^25^. Rough durations of megacontinent/supercontinent pair of Nuna/Columbia and Gondwana/Pangea are based on^25^. Stars indicate the metamorphic rock record marking the final assembly of the supercontinent Columbia at 1.6 Ga. This compilation is superimposed to a histogram and probability curve for the metamorphic age record.

The formation of the first stable cratons and the emergence of plate tectonics were vital steps in the transformation of Earth from the primitive beginning towards the habitable planet of the Phanerozoic. The timing of the onset of plate tectonics has been a subject of intense debate with studies proposing its existence as far back as the Hadean eon (> 4 Ga)^1^, whereas others argue that it did not appear until the Neoproterozoic era (c. 1–0.5 Ga), less than one billion years ago^2^. One obvious reason for this discrepancy of when plate tectonics initiated is using single proxies to discriminate between different tectonic regimes and the natural poor preservation of the ancient high-pressure metamorphic rock record as indicative for the existence of a global plate mosaic, which provides realistic forces to drive mantle convection with deep subduction systems^1^. The oldest lines of evidence for such a global subduction network were suggested using seismic data from six continents at ca. 2 billion years (Ga), coinciding with the formation of the oldest supercontinent on Earth known today, Nuna-Columbia^3^. This aligns with published metamorphic thermobaric ratios and paleomagnetic data that demonstrate the activity of plate tectonics on Earth during the time of formation of the oldest supercontinent on Earth^4–6^. Metamorphic rocks result from the chemical and physical transformations of rocks primarily due to variations in pressure (*P*) and temperature (*T*). Consequently, these rocks serve as valuable indicators for studying processes such as crustal thickening, heating, exhumation, and cooling and offer crucial insights into the tectonic environment prevailing at the time of their formation^7^. The thermobaric ratio (*T/P*) has been previously used to distinguish three types of metamorphism^8^ (Fig. 1): high- (*T/P* > 775°CGPa^−1^), intermediate- (*T/P* = 775–440°CGPa^−1^), and low- (*T/P* < 440°CGPa^−1^) *T/P* metamorphism, where the revised low-*T/P* thermobaric ratio was considered^9^. Previous attempts to integrate paleogeographic reconstructions with the metamorphic rock record have revealed a correlation between low-, intermediate- and high-*T/P* ratios and zones of subduction, mountain building and intracontinental orogens or back-arcs, respectively^10^. Mainly three distinct geodynamic phases were identified using the global metamorphic rock record^8^ (Fig. 1). Phase I (> 2.3 Ga) witnessing the assembly of continental blocks into supercratons^11^, which have been shown to be either short lived or by far smaller than supercontinents^10^; Phase II (2.3–0.7 Ga) characterizing a sharp increase of thermal gradients with dominant high-*T/P* metamorphism. Phase III (> 0.7 Ga) showing a steep decline of thermal gradients towards an all-time low and the appearance of widespread low-*T/P* metamorphism before a subsequent increase and repeated decline related to Pangea’s cycle. Brown and Johnson^8^ relate the evolution of metamorphic thermal gradients with the onset of plate tectonics, with the appearance of metamorphism within localized Neoarchean subduction and a transition towards modern-style plate tectonics with the onset of deep and cold subduction in the Neoproterozoic. However, the ambiguous significance of rare low-*T/P* metamorphic occurrence in the Paleo- and Mesoproterozoic was interpreted to reflect first but obscure evidence of cold subduction potentially related to local anomalies, therefore not relatable to a global evolution. Reconstruction of paleo-positions for the metamorphic rock record for the past 2 Gyr highlights that Paleo- to Neoproterozoic orogens spanning the Columbia and Rodinia supercontinent cycles were much hotter^12^ compared to Phanerozoic times and that Proterozoic low-*T/P* occurrence were notably located at the periphery of the assembling supercontinent^10^. Nonetheless, no local versus global tectonic implications of this Paleoproterozoic, unusually low-*T/P* metamorphism were discussed, while the preservation of these relicts reflect the oldest known occurrence of a potentially colder and deeper subduction environment compared to the Archean, more like the Phanerozoic subduction systems. Therefore, in this contribution we address this crucial open question by analyzing the distribution of the oldest low-*T/P* metamorphic rocks coupled with intermediate-*T/P* versus the occurrence of only intermediate- to high-*T/P* in the Columbia paleogeographic frame.

### Paleogeographic distribution during the assembly of Columbia

**Figure 2:**
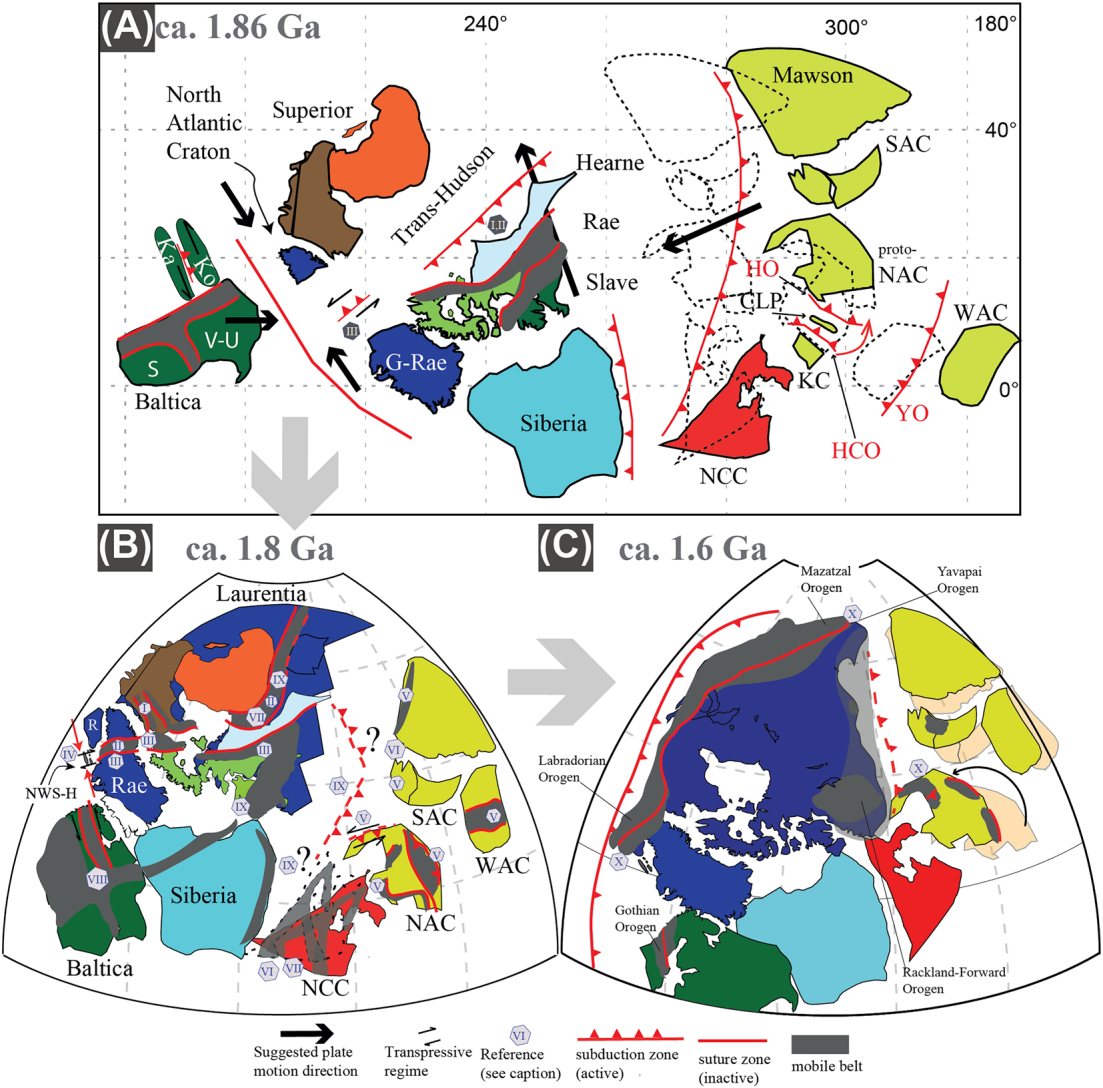
Nuna paleogeographic reconstructions at (**A**) ca. 1.86 Ga, (**B**) ca. 1.8 Ga, and (**C**) ca. 1.6 Ga. Large grey arrows show the temporal succession. Smaller bold black arrows suggest tectonic motion, with smaller curved arrow in the 1.6 Ga reconstruction indicates the rearrangement of the North Australian Craton relative to Laurentia^24^. An alternative position of the North China Craton is also shown, which is not supported by paleomagnetic data^3^. Dotted continent outlines in (A) indicate proposed accompanying motions after the assembly of NAC. Abbreviations for Baltica: *S* Sarmatia, *V-U* Volgo-Uralia, *Ka* Karelia, *Ko* Kola. Greenland (in blue) *G-Rae* Greenland-Rae Craton, *R* Rockall, *NWS-H* NW Scotland and Hebrides. China (in red): *NCC* North China Craton. Australia (in yellow): *proto-NAC* proto-North Australian Craton, *CLP* Central Lamboo Province, *HO* Hooper Orogeny, *HCO* Halls Creek Orogeny, *YO* Yampi Orogeny, *WAC* West Australian Craton, *SAC* South Australian Craton. References for tectonic boundaries and structures: (I) Cutts and Dyck^29^; (II) Corrigan et al.^26^; (III) St-Onge et al.^27^ and Harrison and St-Onge^73^, Kolb et al.^74^, Buchan et al.^35^; (IV) Park^75^; (V) Betts et al. (2015); (VI) Brown et al.^62^, Zhang et al.^76^, Wan et al.^3^; (VII) Chen et al.^77^; (VIII) Buchan et al.^35^, Lathinen et al.^78^; (IX) Perhsson et al.^18,28^; (X) Furlanetto et al.^51^.

There is still uncertainty about the paleogeographic evolution between 2.0 and 1.6 Ga leading to the assembly of Earth’s first supercontinent Columbia (Fig. 2)^5,13,14^. The amalgamation of continental blocks between 2.0 and 1.8 Ga generated global-scale Paleoproterozoic mobile belts^15^. The global zircon age record indicates clear continuous crustal growth and orogenic activity from 1.8 until 1.6 Ga^16^, although subduction- and collisional-type metamorphism shows a bimodal distribution holder with major peaks between 2.0 and 1.8 Ga and minor peaks at 1.6 Ga^17^ (Fig. 1). Paleomagnetic data for a detailed reconstruction of Columbia is also still inconclusive^6,18,19^. Recent metamorphic and structural studies suggest that Columbia’s final assembly occurred only after the collision between NW Laurentia (North America) and proto-Australia at c. 1.6 Ga^20–22^. Indeed, recent detailed comparisons of paleomagnetic data propose a two-stage assembly of Australia and Laurentia at 1.8 (i.e., Nuna) and at 1.6 Ga (i.e., Columbia). This is agreement with existing tectonic models and geological records^23^ and aligns with the proposed combination of a generally valid megacontinent-supercontinent pairing during all supercontinent cycles (Figs. 1 and 2), where a megacontinent reflects several continents assembled during an initial amalgamation stage over the subduction girdle of the previous supercontinent, geodynamically linked to the subsequent formation of the next supercontinent^24,25^. Nonetheless, open questions remain unanswered including: (i) are the first Paleoproterozoic low-*T/P* metamorphic rocks associated with localized subduction-like processes, or do they mark the onset of down-going slabs associated with first global subduction networks? (ii) If the latter is true, why have not more high-pressure relicts been observed within the widespread Proterozoic orogenic belts and why are they potentially located on the edges of the supercontinent?^10^ (iii) Was the formation of the first supercontinent Columbia characterized by a two-stage assembly where the first bimodal metamorphic belts were generated? To address these questions, we combine the metamorphic rock record from 2.0 to 1.6 Ga in a continental paleogeographic reference frame and show its relationship with the supercontinent Columbia to discuss the two-stage amalgamation of the oldest supercontinent and the significance of the low-, intermediate-, and high-*T/P* metamorphism distribution in terms of tectonic regimes. To do this, we compare the tectono-metamorphic evolution at 1.8 and 1.6 Ga for two separated regions within the supercontinent Columbia. The first region is a portion of the Nagssugtoqidian Orogen, where low- and intermediate-*T/P* metamorphic rocks are preserved at ca. 1.85 Ga, namely NW Scotland. Secondly, we present data from NE Australia, where intermediate- to high-*T/P* metamorphism is dominant at ca. 1.6 Ga.

## Tectono-metamorphic evolution during Nuna–Columbia

### Stage I accretion (2.0–1.8 Ga)

#### North Atlantic Craton in Laurentia

**Figure 3:**
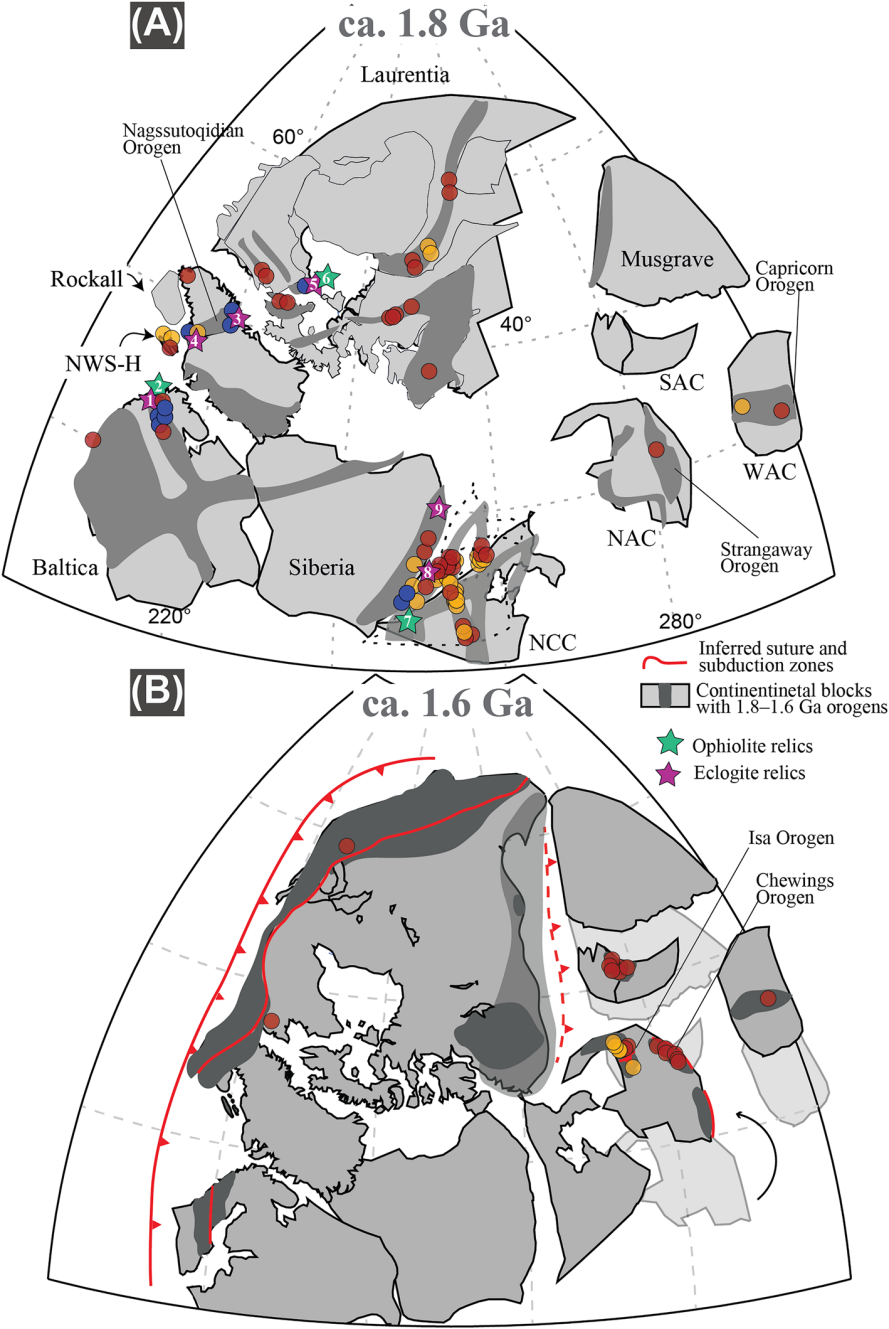
Synoptic reconstruction of the two-stage assembly of Columbia with superimposed orogens and metamorphic rock record at (**A**) 1.8 Ga and (**B**) 1.6 Ga. Green and purple stars reflect ophiolite and eclogite relics, respectively, reported by Wan et al.^3^. Numbered references: (1) Yu et al.^65^, (2) Kontinen^66^, (3) Glassley et al.^31^, and Willigers et al.^32^, (4) Müller et al.^30^, (5) Weller and St-Onge^33^, (6) Scott et al.^43^, (7) Wan et al.^70^, (8) Xu et al.^69^, (9) Gornova and Glazunov^68^. Red, orange, and blue circles represent the metamorphic rock record between 2.0 and 1.6 Ga as reported in Brown and Johnson^71^. Small curved black arrow indicates relative motion of the North Australian Craton with respect to Laurentia (see Fig. 2).

Metamorphic peaks between 2.0 and 1.8 Ga (Fig. 1) reflect global-scale formation of wide orogenic systems. The Trans-Hudson Orogen in the core of proto-Laurentia followed accretion and collision of the Superior, Hearne, Rae, and Slave cratons^26^, whereas the Nagssugtoqidian and Lapland-Kola mobile belts^27,28^, generated after convergence and collision between the Rae and the North Atlantic cratons, and between the Kola and Karelia cratons, respectively (Figs. 2 and 3). The western part of Laurentia started to amalgamate as early as ca. 1.95 Ga and recorded protracted amphibolite- to granulite-facies metamorphism associated with the final amalgamation of the Slave and Rae cratons^29^. Crustal accretion and growth along the Laurentian margin continued until the final collisional event at ca. 1.8 Ga with the Trans-Hudson Orogen, suturing eastern Laurentia with the Superior Craton to form proto-Laurentia^28^. On the eastern side of proto-Laurentia, at ca. 1.9 Ga, the Rae, North Atlantic, Kola, and Karelia cratons were isolated continental blocks moving relative to each other due to active subduction and accretion along their active margins (Fig. 2). Subduction activity is exemplified by calc-alkaline magmatism at ca. 1.92–1.86 Ga and ultra-high pressure (UHP) eclogite and high-pressure (HP) amphibolite-facies relicts at ca. 1.88 Ga^30^, 1.85 Ga^31,32^, and 1.83 Ga^33^ (Fig. 2). The N–S-oriented convergence of the Rae and the North Atlantic cratons, acting as lower and upper plate environments, respectively, resulted in the formation of the E–W-striking Nagssugtoqidian orogenic system at c. 1.85 Ga^34,35^, recorded by collisional-type metamorphism at medium to upper amphibolite-facies^36^. During this time, the Archean Lewisian Gneiss Complex (LGC) in NW Scotland reflected the eastern extension of the Nagssugtoqidian orogenic system, bounded by proto-Laurentia to the west and Baltica to the east^35^. In the Laxfordian (ca. 1.95–1.65 Ga) LGC, subduction-related calc-alkaline granitic intrusions at ca. 1.9 Ga^37^ were regarded as affine to the arc magmatism recorded in the eastern portion of the Nagssugtoqidian^36^. Also, ca. 1.87 Ga HP granulite from the Harris Granulite Belt in the Outer Hebrides^38^ represents buried crustal segments of the accreted margin. This first convergence stage was followed by thickening and burial of the southern portion of the LGC to ca. 30 km. During this collisional stage, ca. 1.85 Ga sub-vertical, NW-striking axial planar fabric of upright folds developed at upper amphibolite-facies reflecting crustal thickening during major NE-directed compression and NW-directed shearing^39^. Synchronous ENE-directed compression coupled with sinistral strike-slip movements between the Kola and Karelia cratons led to the Lapland-Kola mobile belt, almost orthogonal to the Nagssugtoqidian Orogen (Fig. 2).

#### Australian cratons

In cratonic Australia, large-scale accretion of continental blocks occurred between ca. 1.86 Ga and 1.8 Ga and was associated with the amalgamation of micro-continents along the western, southern, and eastern margins of the proto-North Australian Craton (NAC; Fig. 2). On the western margin, the initial accretion of the Kimberly Craton with the Lamboo Complex magmatic arc at 1.86–1.85 Ga formed the Hooper Orogeny, whereas the final collision with proto-NAC occurred during the Halls Creek Orogeny at ca. 1.83–1.8 Ga (Fig. 2)^4,40^. South-dipping subduction of the proto-NAC underneath the Arunta Inlier started at ca. 1.85 Ga and is exemplified by the Willowra and the Leibig Suture zones^23,41^, later reactivated during north-dipping subduction south of the Arunta Inlier at ca. 1.7–1.65 Ga^42,43^. Between 1.9 to 1.85 Ga the eastern margin of the proto-NAC experienced the Barramundi Orogeny, which was characterized by the development of intense N–S striking gneissic axial planar fabric of upright folds during major E–W crustal shortening^44^. The latter was attributed by some authors to be the result of intraplate orogenesis^45^, whereas others suggested accretion of the Isa terrane at ca. 1.87 Ga to the eastern margin of the proto-NAC or of other terranes (e.g., Numil Terrane) at ca. 1.81 Ga suturing a small ocean along the Gidyea Suture^23^. Nonetheless, during this time of accretion, no HP rocks nor ophiolitic remnants were identified within the Australian orogenic belts. In contrast, upper amphibolite- to granulite-facies rocks reflecting medium- to high-*T/P* metamorphism were dominant, indicating crustal thickening and intense reworking.

### Stage II accretion (1.8 and 1.6 Ga)

#### North Atlantic Craton in Laurentia

Between 1.8 Ga and 1.6 Ga the Laurentian blocks, including Greenland, were dominated by wide accretionary orogens recording protracted crustal growth and reworking. After the ca. 1.85 Ga Trans-Hudson Orogen, the Laurentian core (present-day North America), underwent a prolonged period of arc terranes accretion at 1.8–1.68 Ga caused by continuous variations in plate convergence rate and directions until 1.65–1.6 Ga^46^. Protracted accretionary history is exemplified by the Grenville Province, where the Labradorian Orogeny (Figs. 2 and 3) included juvenile arc magmatism and low-pressure, high-temperature metamorphism from 1.71 until 1.6 Ga^46^. In the southern portion of Greenland continuous calc-alkaline magmatism persisted until ca. 1.75 Ga during the Ketilidian Orogen^47^, whereas in NW Scotland, the LGC recorded ca. 1.78–1.73 Ga sub-vertical, NW-striking fabrics at upper amphibolite-facies^39^. The structural and metamorphic evidence in the LGC indicates, therefore, continuous burial and thickening during dominant NW-directed shearing and NNE-compression, likely related to continuous transpressional tectonic regimes with the LGC sandwiched between Baltica and the North Atlantic Craton^39^ (Figs. 2 and 3). Syn- to late-collisional granitic sheets at 1.79 Ga and 1.75 Ga formed by partial melting of the upper amphibolite-facies mid-crust. Whereas post-collisional granite sheets and pegmatite at ca. 1.7–1.65 in the LGC and Greenland were associated with residual post-magmatic hydrothermal fluids that circulated along crustal-scale shear zones at ca. 1.67 Ga^30,48^.

#### Australian cratons

After 1.8 Ga most of Australia was assembled by a combination of continent–continent collision and accretionary events. This amalgamation stage was followed by an extensional phase controlled by an external north-dipping subduction girdle during slab rollback, causing transitional shortening phases within a dominant extensional regime^23^. For example, while deformed granulite- to amphibolite-facies rocks and associated intrusions formed in the Arunta Inlier at ca. 1.74–1.69 Ga during the Strangway Orogeny, localized compression in the Mount Isa Inlier generated basin inversion at c. 1.74 Ga^49^. Nevertheless, from 1.79 Ga to ca. 1.65 Ga tectonic regimes across the NAC were dominantly extensional associated with the development of superbasins such as in the Mount Isa Inlier or the McArthur basins. At this stage, Australia was bounded by a west-dipping subduction zone to the east where a small ocean was separating the eastern margin of the NAC from NW Laurentia^50,51^. During this time, the sedimentary basin of the Georgetown Inlier started to form either as an intracontinental basin as an outboard component of the Mount Isa Inlier^52^, or as a continental ribbon that separated from the NW Laurentian margin at ca. 1.68 Ga^53^. Between 1.69 Ga and 1.66 Ga mafic magmatism and volcanism occurred across different Proterozoic inliers, including the Arunta Inlier^54^, the Mount Isa Inlier and the Georgetown Inlier^55^. Consumption of the oceanic crust between Australia and Laurentia started at ca. 1.7 Ga along the Laurentian margin with eastern Australia as a passive margin until continental collision at ca. 1.65–1.6 Ga^21,22,53^. Several locations have been proposed for the final suture between Australia and Laurentia, remaining this a matter of current debate^20,52,56^. Nonetheless, at 1.6 Ga E–W-directed collision between the Laurentian and Australian blocks formed a N–S-striking compressional fabric recording Barrovian-type metamorphism reflecting burial and thickening of the orogenic system^21^. This stage was followed by an extensional post-collisional stage recording low-pressure, high-temperature metamorphism and related to syn-kinematic magmatism^56–58^. Hence, intermediate- to high-*T/P* metamorphism characterized Australian Mesoproterozoic orogenic belts formed during the 1.6 Ga final Columbia assembly and no HP metamorphic rocks nor ophiolitic remnants were reported.

## Discussion

### Two-stage accretion (2.0–1.8 and 1.8–1.6 Ga) during Nuna-Columbia

The two sample regions considered here offer the opportunity to compare distinct and diachronous metamorphic evolutions of Archean crustal blocks during the slow and prolonged Proterozoic amalgamation of the supercontinent Columbia.

During the first-stage of Nuna assembly (2.0–1.8 Ga)^24^, a long-lasting convergent regime in the Laurentian core, including Greenland, lead to major crustal growth and reworking along active margins through continent–continent collisional events. Within these tectonic setting, oceanic and crustal slivers were buried to great depth and partially preserved as exhumed relics along tectonic boundaries. Their preservation together with bimodal metamorphism along Paleoproterozoic orogenic belts, reflecting typical lower and upper plate environments, are typical components indicative of a mobile-lid regime (Fig. 1). In contrast, between 2.0 and 1.8 Ga the South and Western Australian cratons were characterized by higher thermal gradients and the development of hot orogenic belts with neither the occurrence of HP rocks nor ophiolitic remnants, such as the 1.82–1.73 Ma Capricorn Orogen in the Western Australian Craton, reflecting an intraplate orogen associated with intermediate- to high-*T/P,* magmatism and late hydrothermal fluids^59,60^.

A transitional period of crustal blocks rearrangement during the second-stage of Columbia assembly^24^ was reported in the Australian rock record. Metamorphic quiescence at ca. 1.7 Ga during intracontinental extension was associated with intrusion of dolerite dykes and basaltic pillow basalts in central and eastern Australia^55^. Extensive granitic magmatism, and granulite-facies metamorphism was recorded around 1.73–1.7 Ga in East Antarctica^61^. This transitional stage was followed by a compressional and transpressional regime at 1.65–1.6 Ga in the North and South Australian cratons, East Antarctica, and western Laurentia^62^. Intracontinental collision and accretionary orogens generated intermediate- to high-*T/P* metamorphism in places such as NE Australia^21^, Arunta Inlier^63^ and SE Australia^64^.

While in Laurentia-Greenland and Australia the 2.0 and 1.8 Ga accretionary evolution was reflected by wide collisional orogenic systems suturing several Archean cratonic blocks, the younger amalgamation stage between 1.8 Ga and 1.6 Ga was rather related to subordinate accretionary orogens and/or intracontinental collisional events. Active subduction processes between Laurentia, Greenland, and Baltica during the first-stage coupled with the exhumation of HP rocks during the convergence stage enabled the preservation of low-*T/P* metamorphic rocks along the wide 2.0–1.8 Ga mobile belts, whereas the Australian and the East Antarctica blocks were potentially underlain by a plume causing high geothermal gradients and triggering the formation of hot collisional belts. On the other hand, reorganization of crustal blocks during the second-stage assembly forming Columbia at ca. 1.65–1.6 Ga generated intermediate-to high-*T/P* metamorphic rocks during subordinate collisional orogens within the Australian blocks and major fluid-enhanced crustal reworking along preexisting crustal-scale shear zones in the North Atlantic Craton within Laurentia.

Despite the scarce occurrence and/or limited preservation of subduction-related relicts in the Paleoproterozoic metamorphic rock record, particularly during the plate convergence that led to the formation of the first-stage of the oldest supercontinent (i.e., the megacontinent Nuna), their rare occurrence support a slow, yet globally encompassing mobile-lid regime. An almost N–S striking subduction front associated with the formation of the Lapland-Kola, Nagtussoqidian and the Trans-Hudson orogens and minor orogenic events in Laurentia preserved the oldest metamorphic rock record of HP rocks^30–33,65^ and ophiolitic fragments^66,67^ on Earth. Another N–S striking suture zone and associated orogeneses are preserved between Siberia and the North China Craton, where HP eclogitic rocks^68,69^ and ophiolitic relics^70^ have been reported. These suture zones between Siberia and the NCC may have been part of a subduction system extending along strike between Laurentia, Australia and East Antarctica (Fig. 3). However, no Paleoproterozoic HP relicts were recorded within these terrains^62^.

The Paleoproterozoic HP and oceanic relict reflect the oldest known occurrence of a potentially colder and deeper subduction environment than ever before more similar to the Phanerozoic. Recent authors located these low-*T/P* metamorphic rocks along plate margins defining a peripherical subduction girdle around the supercontinent Columbia^10^. In contrast, our more detailed paleogeographic reconstruction shows that bimodal metamorphism characterized by low- and intermediate- to high-*T/P* metamorphism was preserved along Paleoproterozoic orogenic belts located in subduction-related environments within the core of the megacontinent Nuna (Fig. 3a). Whereas the second-stage amalgamation forming the supercontinent Columbia reflected localized rearrangement of crustal blocks producing intermediate-*T/P* metamorphic rocks during soft collisions and high-*T/P* metamorphism mostly during late- to post-collisional stages.

Overall, this contribution clearly emphasizes the link between the preserved bimodal metamorphic rock record and the paleogeographic reconstruction of major crustal blocks and plate margins during the Paleoproterozoic (Fig. 3). The present integration of thermobaric ratios with paleogeographic data shows localized metamorphic bimodality during the first-phase accretion of Nuna (2.0–1.8 Ga) and a dominant intermediate- to high-*T/P* metamorphism during the second-phase of final assembly of the supercontinent Columbia (1.8–1.6 Ga). The oldest low-*T/P* metamorphic rocks are localized along mobile belts forming a main suture in the core of the assembling megacontinent Nuna. Accordingly, our results may be interpreted as the beginning of a mobile-lid mode stage much like plate tectonics leading to the formation of Earth’s first supercontinent Columbia, where a larger scale subduction network like the Phanerozoic started to operate.

Interestingly, the most recent supercontinent Pangea witnessed a contrasting evolution compared to the older Columbia supercontinent. The assembly of the Gondwana megacontinent was characterized by fewer instances of intermediate and low-*T/P* metamorphism, whereas the subsequent Pangea assembly involved widespread production and exhumation of HP and UHP metamorphic rocks along pronounced orogenic systems such as the Appalachian-Variscan mountain belt^10^. This divergence in evolution may have been related to factors such as i) the size of the colliding continental blocks and ii) the thermal gradients established along the convergent margins. This difference in the metamorphic record highlights that the Nuna megacontinent assembly involved significant continent–continent collisions, possibly resulting in the development of colder geothermal gradients within these settings. This was followed by intracontinental rearrangements and intraplate magmatism during the formation of the Columbia supercontinent. In contrast, the Pangea supercontinent assembly reflected widespread and major continent–continent collisions, leading to the formation of a substantial amount of low-*T/P* metamorphic rocks (Fig. 1). Overall, the comparison of the Columbia and Pangea supercontinent assemblies reveals that, although both assemblies can be divided into two stages, a closer examination shows fundamental tectonic differences between their evolutions, potentially linked to the initiation of the supercontinent cycle in the Paleoproterozoic.

## Methods

### Metamorphic database

The global dataset of the metamorphic rock record used in this contribution was retrieved from Brown and Johnson^71^ and integrated with published literature, compiled in the reference list of the Supplementary Table S1, for the timespan between ca. 2.0 and 1.6 Ga. The data compiled in Table S1 are divided in three groups: (i) High-*T/P* metamorphism, (ii) Intermediate-*T/P* metamorphism and (iii) Low-*T/P* metamorphism. The boundary between the intermediate/high-T/P thermobaric ratios were maintained as proposed by Brown and Johnson^71^, whereas the revised value of 440 °C/GPa was used for the low- to intermediate-T/P transition^9^. The reason for using the more recently revised thermobaric ratios value for this transition is that 440 °C/GPa more closely reflect the thermodynamic modelling and experimental results obtained for high-pressure granulite to eclogite of mafic Precambrian rocks^9^. For all data pressure, temperature, metamorphic age (Ga), and location is reported. For latitude and longitude of the data added to the already existing global dataset^71^ the reader may refer directly to the associated publication. The *P–T* estimates reported may be an average based on multiple samples analyzed by the same study. For additional information on the method for data acquisition and compilation see^71^.

### Paleogeography

For the paleogeographic reconstructions the broad setup of Kirscher et al.^4^ was used for Laurentia, Siberia, Australia and North China cratons with some slight modifications (Supplementary Fig. S1). Recent paleomagnetic compilations were used to reconstruct the position of Baltica^72^. We still acknowledge some uncertainty concerning the position of the North China Craton relative to Siberia, where paleomagnetic data rather suggest a separation of the two, whereas geological evidence suggest a closed configuration^3^ (Supplementary Table S2).

### Supplementary Information

Supplementary Figure S1.

Supplementary Table S1.

Supplementary Table S2.

Supplementary Legends.

## Data Availability

All data generated or analyzed during this study are included in the Supplementary Information files of the published article [Global dataset of metamorphic rock record modified after Brown and Johnson^71^].
