# Supplementary figures and images for: Metamorphic turnover at 2 Ga related to two-stage assembly of Columbia
Source: Sci Rep. 2024 Mar 18;14:6483. doi: 10.1038/s41598-024-56691-1 (PMC10948810; doi:10.1038/s41598-024-56691-1)

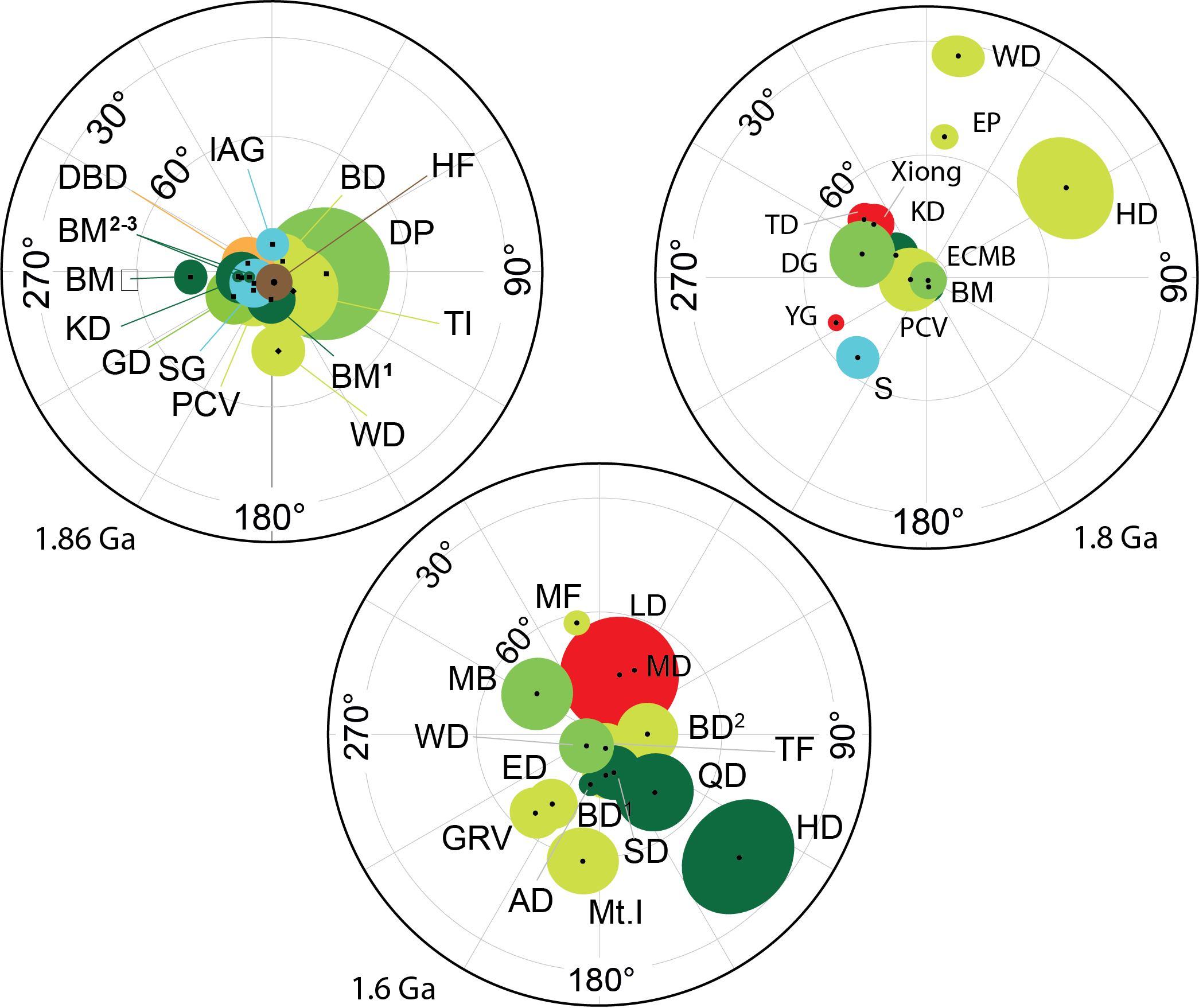

Supplement: Supplementary file 1 — Supplementary Figure S1. [file 41598_2024_56691_MOESM1_ESM.png]
