# Supplementary material for: Metamorphic turnover at 2 Ga related to two-stage assembly of Columbia
Source: Sci Rep. 2024 Mar 18;14:6483. doi: 10.1038/s41598-024-56691-1 (PMC10948810; doi:10.1038/s41598-024-56691-1)
Supplement: Supplementary file 4 — Supplementary Legends. [file 41598_2024_56691_MOESM4_ESM.docx]

Metamorphic turnover at 2 Ga related to two-stage assembly of Columbia

Silvia Volante*^1^ & Uwe Kirscher^2^

^1^ Structural Geology and Tectonics Group, Geological Institute, Department of Earth Sciences, ETH Zürich, svolante@ethz.ch

^2^ Earth Dynamics Research Group, The Institute for Geoscience Research (TIGeR), School of Earth and Planetary Sciences, Curtin University, Western Australia, Australia, uwe.kirscher@curtin.edu.au

Caption Supplementary Figure

Fig. S1 Paleomagnetic poles based on the reconstruction used in this study for 1.86, 1.8, and 1.6 Ga. Paleopoles are shown with respective confidence ellipses (A95), which are color coded by craton where the paleomagnetic data is based on: Red— North China Craton, light blue—Siberia, dark green— Baltica, green— Laurentia, light green— Australia. Pole abbreviation are related to supplementary Table S2.
